# DisCP-Atlas: a comprehensive resource mapping cellular processes to complex diseases

**DOI:** 10.1093/nar/gkaf1129

**Published:** 2025-11-03

**Authors:** Frank Qingyun Wang, Caicai Zhang, Hangchen Zhang, Xiao Dang, Yanqun Sun, Siyuan Bu, Xu Wang, Wanling Yang, Chen Cao

**Affiliations:** Department of Paediatrics and Adolescent Medicine, The University of Hong Kong, Hong Kong 999077, China; Department of Paediatrics and Adolescent Medicine, The University of Hong Kong, Hong Kong 999077, China; Jiangsu Key Laboratory for Biomedical Electromagnetic Precision Theranostics, School of Biomedical Engineering and Informatics, Nanjing Medical University, Nanjing, Jiangsu 211166, China; Department of Paediatrics and Adolescent Medicine, The University of Hong Kong, Hong Kong 999077, China; Clinical Medical Research Center, Children’s Hospital of Nanjing Medical University, Nanjing, Jiangsu 210008, China; Department of Neurology in Affiliated ZhongDa Hospital and Jiangsu Provincial Medical Key Discipline, School of Medicine, Institution of Neuropsychiatry, Key Laboratory of Developmental Genes and Human Disease of Ministry of Education, Southeast University, Nanjing, Jiangsu 210009, China; Clinical Medical Research Center, Children’s Hospital of Nanjing Medical University, Nanjing, Jiangsu 210008, China; Department of Paediatrics and Adolescent Medicine, The University of Hong Kong, Hong Kong 999077, China; Jiangsu Key Laboratory for Biomedical Electromagnetic Precision Theranostics, School of Biomedical Engineering and Informatics, Nanjing Medical University, Nanjing, Jiangsu 211166, China

## Abstract

Genome-wide association studies (GWAS) have identified thousands of disease-associated loci, yet translating these findings into biological mechanisms remains challenging. Single–cell RNA–sequencing provides functional annotation at cellular resolution. Most current resources connect disease associations only to discrete cell–type categories. This cell type centric view overlooks disease-relevant cellular processes, which constitute the primary gene expression programs underlying genetic risk. These cellular processes function along continuous cell-state trajectories and routinely extend beyond conventional cell type boundaries. To address this issue, we present DisCP-Atlas (Disease to Cellular Process Atlas), a comprehensive resource that maps cellular processes to complex diseases. DisCP-Atlas identifies 990 cellular processes derived from 57 single-cell datasets across 35 human tissues, each annotated based on pathway enrichment and cell type-specific activities. Leveraging the sc-linker framework, we systematically linked 1072 GWAS datasets spanning 759 diseases to the cellular processes and identified 37 918 significant cellular process–disease associations. DisCP-Atlas allows bidirectional querying diseases to their underlying cellular processes or identifies diseases associated with specific cellular programs. All the results are presented in interactive visualization and can be conveniently downloaded. Additionally, DisCP-Atlas provides an online enrichment tool, enables users to submit custom genes, and returns detailed tables and interactive visualizations of enriched cellular processes. DisCP-Atlas is freely accessible at https://www.discpatlas.net.

## Graphical Abstract

**Graphical Abstract:**
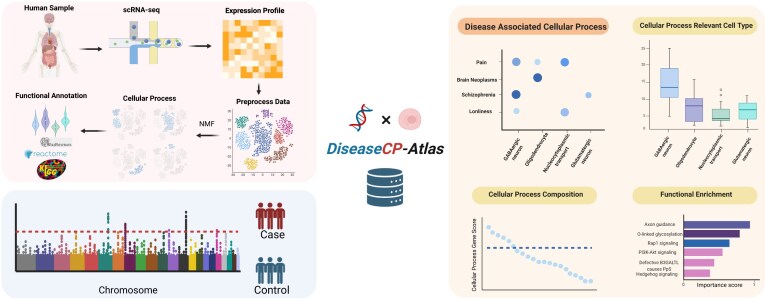

## Introduction

Genome-wide association studies (GWAS) have identified numerous associated variants over the past few years, greatly improving our understanding of human complex traits [1–4]. However, the cellular mechanisms underlying how these genetic variants influence complex traits remain elusive [1, 5]. A significant portion of the identified variants is located within noncoding regions, where they act as regulators of distant genes [6, 7]. Furthermore, these variants are shown to have context-specific regulatory functions, making it challenging to determine the specific cell types and cellular processes in which they operate [8]. The recent advancements in single-cell technology have vastly improved our understanding of the biological functions at the cellular level, offering an important resource to help us better understand the GWAS findings [9].

Previous computational methods have been developed to connect single-cell transcriptomic data with GWAS findings. The CELLECT computational framework aims to identify the relevant cell types responsible for complex traits by creating cell type-specific expression profiles and linking them to GWAS results [10]. On the other hand, single-cell Disease Relevance Score (scDRS) utilizes a permutation approach to pinpoint individual cells that exhibit overexpression of a set of genes identified through MAGMA analysis [11]. Building on these efforts, several knowledge bases have emerged to curate the correlation between single-cell RNA-seq data and GWAS traits. SC2disease links cell types to diseases by overlapping cell type-specific genes with genes associated with lead single nucleotide polymorphism (SNP), albeit lacking statistical robustness [12]. CSEA-DB can prioritize GWAS genes in established cell types but may overlook heterogeneity within subpopulations [13]. More recently, SC2GWAS has leveraged scDRS to identify individual cells overexpressing disease-associated genes identified by MAGMA, addressing population heterogeneity and improving the analysis to cellular resolution [14].

While existing resources provide valuable insights, their reliance on discrete clustering and marker-based annotation presents a critical limitation. These approaches often fail to capture the dynamic, cross-boundary nature of disease-relevant cellular processes, which frequently operate along continuous cell states and transcend conventional cell type classifications [15]. Recognizing the significance of the cellular process, sc-linker [16] has been increasingly utilized to dissect cellular processes across heterogeneous immune, endothelial, neuronal, and other cell populations. This strategy has successfully uncovered pathogenic cellular processes associated with a wide range of complex diseases, including autoimmunity, cardiovascular, and psychiatric diseases, among others [17–19]. This paradigm shift toward cellular process-centric analysis provides a more biologically relevant framework for uncovering continuum-driven molecular programs, understanding disease pathogenesis and offering novel therapeutic opportunities that are inaccessible through conventional approaches relying on rigid cell type categorization.

**Figure 1.**
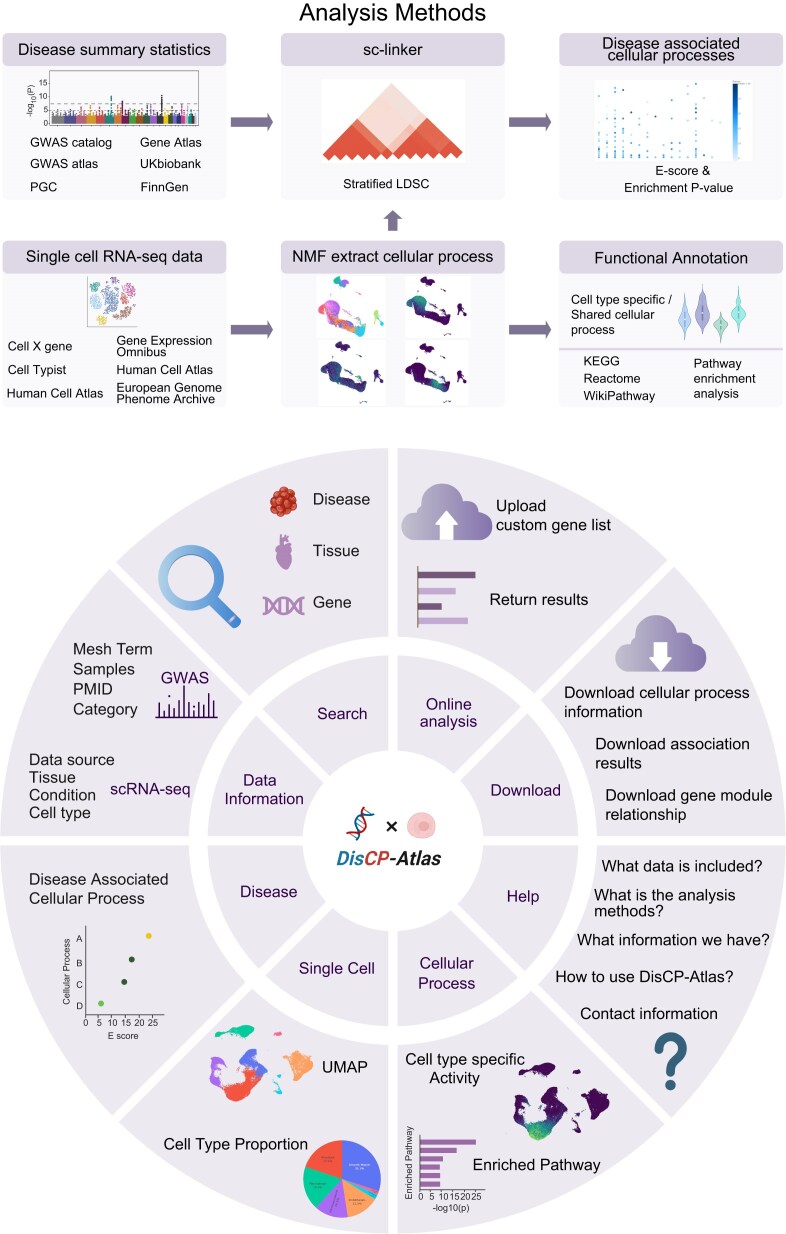
Overview of the DisCP–Atlas workflow. The upper panel illustrates the analytical workflow implemented in DisCP-Atlas. ScRNA-seq datasets from various public repositories (CellXgene, Gene Expression Omnibus, Human Cell Atlas, European Genome-Phenome Archive) were processed to identify cellular processes via consensus non-negative matrix factorization (cNMF). GWAS summary statistics from multiple databases (GWAS Catalog, Gene ATLAS, GWAS ATLAS, UK Biobank, PGC, FinnGen*)* were analyzed using stratified LD-score regression (S-LDSC) within the sc-linker framework to identify disease-associated cellular processes. Cellular processes were further annotated for cell type specificity and biological pathways (KEGG, Reactome, WikiPathways), with enrichment scores (*E*-scores) and *P*-values provided. The lower panel describes the interactive user interface of DisCP-Atlas, structured around six key modules Disease, Single Cell, Cellular Process, Data Information, Online Analysis, and Download. Users can query diseases, tissues, genes, or cellular processes, explore detailed metadata, visualize single-cell data through UMAP embeddings and cell type proportions, perform customized enrichment analyses, and download comprehensive data tables and results for further investigation.

Here, we present DisCP-Atlas (Disease to Cellular Process Atlas), which identifies 990 cellular processes represented by gene expression modules and connects them with 1072 GWAS summary statistics spanning 759 diseases, yielding 1 061 280 cellular process–disease associations (Fig. 1). To improve biological interpretation, we annotate these cellular processes by examining their cell type specificity and performing functional enrichment analysis. The web interface offers interactive plots for browsing the process catalog and associated diseases, together with an online enrichment tool. DisCP-Atlas therefore provides a unified platform for querying, visualizing, and downloading cellular processes derived from single-cell RNA sequencing (scRNA-seq) data and their relationships to complex diseases.

## Materials and methods

### GWAS summary statistics collection and processing

We manually collected GWAS summary statistics from several publicly available repositories. The UK Biobank GWAS summary statistics were obtained from Neale Lab (http://www.nealelab.is/uk-biobank), Gene ATLAS [20], and GWAS ATLAS [21]. Additional GWAS summary statistics were downloaded from the GWAS Catalog [22], GRASP [23], and PGC (https://pgc.unc.edu/).

Quality control of the GWAS summary statistics followed a similar rigorous, standard pipeline described in previous studies [24, 25]. Briefly, we removed the datasets without the essential information, such as sample size or population information. We also excluded datasets with missing or incorrect data, such as those without standard rsIDs or beta values. Then, we standardized the reported diseases by matching them to MeSH terms to avoid confusion.

To ensure robust genetic correlation estimates between diseases and cellular programs and maintain relevance to the single-cell dataset, we restricted our analysis to diseases classified as complex diseases or mental/behavioral disorders. Finally, we obtained 1072 GWAS summary statistics of European ancestry spanning 759 diseases from nearly 8000 publicly available datasets, with 41% of the traits supported by multiple GWAS summary statistics (Supplementary Table S1).

SNP-level filtering was then performed following the previously established standard protocols [24–28]. Briefly, we excluded SNPs with minor allele frequency (MAF) <0.01 or duplicate identifiers. We excluded datasets lacking essential fields, including rsIDs, beta coefficients, or *Z*-scores, unless imputation was feasible. For partially incomplete datasets, we imputed *Z*-scores when appropriate. We used the LiftOver tool to convert GRCh38-based data to GRCh37 [29]. When MAF data were missing, we substituted MAF values from the European-ancestry panel of the 1000 Genomes Project [30].

### Single-cell RNA-seq data collection and processing

We curated human scRNA-seq datasets from public repositories, including Gene Expression Omnibus [31], CELLxGENE [32], Human Cell Atlas [33], CellTypist [34] retaining studies that contained >10 000 cells and covered a broad range of human tissues. This procedure yielded 57 scRNA–seq datasets spanning 35 tissues for downstream analysis (Supplementary Table S2). In this initial version of DisCP-Atlas, our primary objective was to curate single-cell datasets encompassing a wide array of tissues, achieving a breadth comparable to large-scale projects such as the Genotype-Tissue Expression (GTEx) project [35]. The inclusion criteria required datasets to contain at least 10 000 cells. Where possible, we incorporated multiple datasets under different conditions for the same tissue type in the current release to facilitate internal replication and enhance robustness.

All datasets were converted to H5AD format and loaded into Scanpy [36]. Cell-level quality control removed cells with <200 or >5000 detected genes and those with mitochondrial gene content ≥20%. When available, we utilized the original UMAP embeddings and cell annotations provided by the authors or the original source. For datasets lacking precomputed UMAP or provided cell type annotation, we conducted standard preprocessing starting with highly variable gene selection using scanpy.pp.highly_variable_genes, followed by technical covariate regression (total counts and mitochondrial percentage) with scanpy.pp.regress_out. We then performed PCA (scanpy.tl.pca, svd_solver=‘arpack’) and applied Harmony batch correction (scanpy.external.pp.harmony_integrate) using sample as a key covariate to address batch effects. The integrated data were used for downstream analysis including neighborhood graph construction (scanpy.tl.neighbors), Leiden clustering (scanpy.tl.leiden), and UMAP visualization (scanpy.tl.umap), with cell type annotation based on canonical markers from the CellMarker database [37]. The manually annotated and provided cell types were then cross-validated with the results derived from the CellTypist results to ensure the accuracy of the annotation [34].

### Identification of the cellular process

Non-negative matrix factorization (NMF) is a robust method to define complex gene expression modules from single-cell RNA-seq data in an unsupervised way without relying on predefined cell type annotations [38, 39]. In this study, we utilized cNMF on the normalized expression matrices of each dataset, retaining genes expressed in >5% of cells [40]. For an input count matrix ${\mathrm{X}} \in {\mathrm{R}}^{{n} \times {g}}$, where *n* is the number of cells and *g* is the number of genes, cNMF returns (i) a matrix ${\mathrm{W}} \in {\mathrm{R}}^{{k} \times {g}}$, whose entries represent the contribution of each gene to each cellular process (or module), and (ii) a matrix ${\mathrm{H}} \in {{{\mathrm{R}}}^{{\mathrm{n}} \times {\mathrm{k\ }}}}$, which describes the usage of each cellular process across individual cells. *K* denotes the number of NMF components, which is the number of cellular processes learned from each single-cell dataset.

Because single-cell transcriptomes are high-dimensional and hierarchical, a single “optimal” *K* is rarely biologically unique. We therefore restricted *K* to 10–20 to balance interpretability and cross-dataset comparability at the tissue level. *K* was selected by jointly evaluating reconstruction error and factorization stability across repeated runs and by inspecting stability versus error plots to locate the region where additional components provided diminishing gains while maintaining adequate stability. Biological interpretability was then confirmed by annotating cell type specificity and by performing pathway enrichment using KEGG, Reactome, and WikiPathways.

The default parameters were used to run the cNMF pipeline on all the single-cell data. To select the optimum number of *K* cellular processes, we used a recommended heuristic approach by selecting the *K* where the largest difference occurs between scaled solution stability and scaled solution error [40]. Rather than selecting purely on the basis of maximal stability, which may bias toward underestimating the number of factors, we focused on the region where the trade-off between reconstruction error and stability was the most informative. This approach highlights the point at which additional components provide diminishing improvements in reconstruction accuracy while maintaining sufficient stability to ensure reproducibility. Applying this procedure to 57 datasets yielded 990 modules, which we treat as cellular processes for downstream analyses.

### Linking cellular processes to the GWAS traits

We quantified the association between each cellular process and each GWAS trait using S–LDSC coupled with the tissue–specific Roadmap–ABC enhancer-gene linking strategy implemented in sc-linker [38, 39]. The method accepts a weighted gene list derived from single–cell RNA–seq. Each weight represents the min–max–scaled contribution of a gene to the cellular process, and it uses S–LDSC to test whether SNPs linked to these genes are significantly enriched for the heritability of a given GWAS trait.

The gene weights for each cellular process were calculated using min–max normalization to process the contribution score of each gene to each cellular process in the ${\mathrm{W}} \in {\mathrm{R}}^{{k} \times {g}}$ matrix. To maximize the specificity of each cellular process, we only retained the top 5% gene weights for the downstream analysis. The LD score for the SNPs annotated to each cellular process was calculated based on the gene weights using the 1000 Genomes Project European population panel [30]. S-LDSC then quantified the proportion of disease trait heritability attributable to each cellular process [41]. The *E*-score was calculated as the difference between the enrichment for an annotation in a specific program and the SNP annotation for all protein-coding genes in the genome. A total of 1 061 280 possible cellular processes to GWAS disease trait pairs are cataloged in the DisCP-Atlas database. We have implemented the Cell Type Ontology Mapper (https://github.com/Starlitnightly/CellOntologyMapper) to integrate standardized cell ontology information into the Standardized Cell Type column on the GWAS page [42]. This enhancement improves cross-study comparability by ensuring consistent cell type classification across different datasets.

### Functional enrichment analysis

To enhance the biological interpretability of the identified cellular processes, we annotated each process based on cell type expression specificity and performed functional enrichment analysis using resources such as KEGG, Reactome, and WikiPathways. Cell type specificity was evaluated with the Wilcoxon rank-sum test that compared the usage of each cellular process in one cell type against all other cells. A significance threshold of Benjamini–Hochberg-adjusted *P*-value <1 *× *10^−5^ and a log_2_ fold change >1 were applied to determine whether the process was overexpressed in a certain cell type.

In order to provide a functional interpretation of each cellular process, we performed functional enrichment analysis using clusterProfiler [43]. We selected the top 5% of genes by weight for each cellular process and converted their symbols to Entrez IDs with the bitr function of clusterProfiler. Functional enrichment analysis was then carried out in clusterProfiler: enrichWP for WikiPathways [44], enrichPathway (ReactomePA) for Reactome [45], and enrichKEGG for KEGG [46]. Pathway terms with an adjusted *P*-value <.05 were retained as significant.

Cellular processes were labeled according to their most specific cell type based on the highest log_2_ fold change value if showing cell type specificity. Processes without clear cell type specificity were labeled with the most significant functional term (lowest adjusted *P*) returned by the enrichment analysis. This step ensures that the selected *K* resulting programs are biologically meaningful, rather than merely technically defined.

### Enrichment analysis of user-provided genes in cellular processes

The web interface allows users to input a gene list and determine the cellular processes in which those genes are most enriched. For each process, the tool computes an observed score, defined as the mean weight of the input genes within that process. Statistical significance is evaluated with a standard permutation test [47]: gene labels are randomly shuffled 1000 times while preserving the number of genes in the list, and an empirical *P*–value is calculated as the fraction of permutations whose score equals or exceeds the observed value. Cellular processes are ranked in descending order of their observed scores, enabling users to prioritize pathways most relevant to their gene set.

### Implementation of the DisCP-Atlas

The backend is implemented in Spring Boot (Java). The frontend is built with Vue and styled with Element UI. We implement interactive visualizations, including UMAP projections and pathway-enrichment plots, with Plotly (Python). All curated data, including single–cell RNA–seq datasets, cellular process–disease association statistics, and GWAS trait metadata, are stored in a MySQL relational database to support fast, query–driven retrieval.

## Results

### DisCP-Atlas statistics

**Figure 2.**
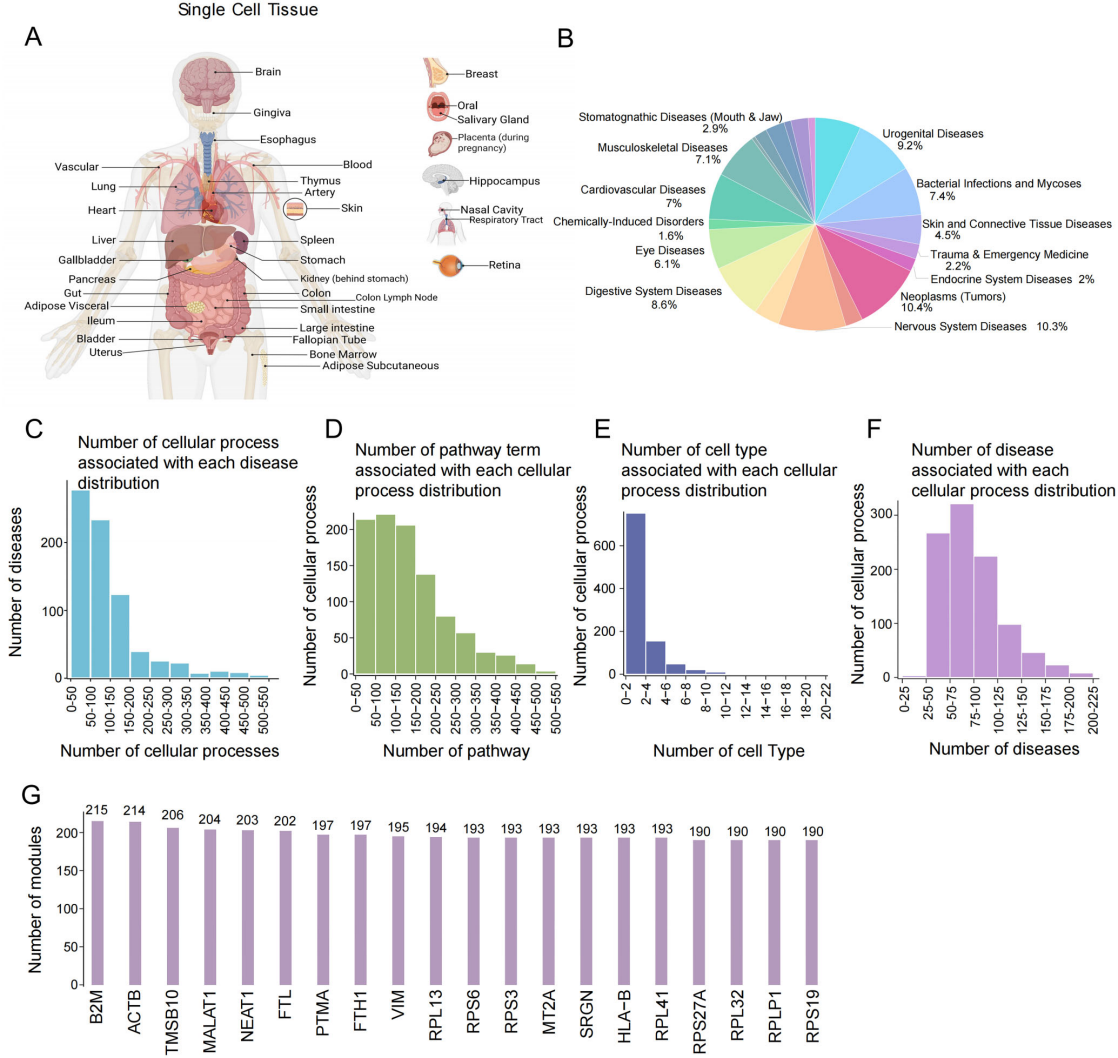
Data statistics for the single–cell datasets, cellular processes, and disease associations in DisCP–Atlas. (**A**) Map illustrating the tissue origins of the 57 single-cell RNA-seq datasets included in DisCP–Atlas. (**B**) Pie chart depicting the proportions of the 21 disease categories defined by MeSH. (**C**) Bar plot showing the number of cellular processes associated with each disease. (**D**) Bar plot illustrating the number of enriched pathway terms (KEGG, Reactome, WikiPathways) per cellular process. (**E**) Bar plot displaying the number of enriched cell types for each cellular process. (**F**) Bar plot showing the number of diseases associated with each cellular process. (**G**) Bar plot listing the top 20 genes involved in the largest number of cellular processes across the atlas.

DisCP–Atlas is a web–accessible resource that identifies cellular processes from single–cell RNA–seq data and links them to complex diseases through curated GWAS summary statistics (Fig. 1). The current release incorporates 1072 European–ancestry GWAS summary statistics spanning 759 diseases drawn from large–scale UK Biobank cohorts and case–control studies, with nearly half of the traits supported by multiple summary statistics. These datasets cover 21 broad disease categories (Fig. 2B), e.g. immune, cardiovascular, and psychiatric disorders. To aid navigation, each trait is cross–referenced to the corresponding terms in the MeSH ontology, enabling users to explore diseases that map to more than one category.

We assembled 57 human single–cell RNA–seq datasets comprising 2481 donors and 35 tissues (Fig. 2A). Processing these data with a uniform pipeline yielded 990 cellular processes. Among them, 919 (92.8 %) were specific to at least one cell type, whereas 71 showed ubiquitous expression within their respective tissues. Most processes were enriched in 0–2 cell types (Fig. 2E). Pathway over–representation analysis assigned 2504 unique WikiPathways, KEGG, and Reactome terms to the processes (Fig. 2D). Collectively, the modules involve 14 860 unique genes and range from 1 to 581 genes in size (median = 376). Genes that appear in the largest number of processes encode core cellular machinery such as ribosomal proteins (RPL/RPS), cytoskeletal components (*ACTB, VIM, TMSB10*), immune mediators (*B2M, HLA–B, MALT1*) and metabolic regulators (*FTL, FTH1, MT2A*) (Fig. 2G). Because housekeeping genes are involved in pervasive cellular processes, they show broad, nonspecific associations in large number of cellular processes with many diseases. Consequently, they often rank as the top genes associated with any disease category (Supplementary Table S4).

Using the sc-linker framework, we evaluated 1 061 280 cellular process–disease pairs and identified 37 918 significant associations (*P*-value <.05). To surface potentially informative signals, we also provide 72 720 associations meeting an exploratory threshold (*E*-score > 1, *P*-value <.1) on the website, while all results remain available for download. On average, each disease is associated with 96 cellular processes (Fig. 2C) and each cellular process is associated with 73 diseases (Fig. 2F), thereby providing comprehensive mechanistic insights into complex diseases. We analyzed two independent GWAS per trait to assess replicability across cohorts with differing ascertainment and pipelines and to increase sensitivity to signals that may be borderline in one dataset yet robust in another when available. For example, for asthma, the association between pulmonary capillary endothelial cell processes (Lung_syn53694312, Module_8) was marginal in one GWAS (*P*-value = .067) but significant in an independent GWAS of the same trait (*P*-value = .044; DisCP0053:2), with consistent effect direction and comparable effect size (*E*-score) across datasets. For inference, the primary significance threshold remains *P*-value <.05. To aid users in exploring potentially informative signals, the web interface additionally displays associations meeting an exploratory threshold of *P*-value <.1, while all procedures and multiple testing controls remain unchanged and full statistics are available for download.

At the disease category level (Supplementary Fig. S1), we demonstrated that cellular processes associated with cardiovascular diseases prominently feature smooth muscle cells from the heart, microvasculature from the vascular system, and astrocytes from the brain. For digestive diseases, the enriched processes include not only mesenchymal-related processes from the colon and enteroendocrine-related processes from the small intestine but also various immune-related cell types, highlighting the significant role of inflammation. As expected, immune-related cellular processes were the most highly enriched for both hemic/lymphatic disease and immune system disease. Notably, smooth muscle cells and vein endothelial cells were also implicated in hemic/lymphatic disease, as these cells facilitate the extravasation of immune cells into tissues and are active. In mental and behavioral disorders, processes related to brain neuron cells were dominantly enriched, strongly aligning with the neurobiological basis of these disorders. In addition, for nutritional and metabolic diseases, the top enriched processes originated from metabolism-related organs, including the small intestine, spleen, and liver, directly connect the molecular findings to the core organs regulating body metabolism. We have also listed a table of cellular processes–disease pairs that align with established biomedical knowledge (Supplementary Table S3).

### Database interface and usage

DisCP-Atlas provides a web interface consisting of six tabs: Single Cell, GWAS, Gene, Module Enrichment, About, Tutorial, and Download. These tabs support bidirectional navigation between diseases and cellular processes. Queries can start from a disease, a tissue/cell type, or a gene. The homepage displays a version number and release date with a simple changelog, so that users can check and cite the exact snapshot of the resource.

In the GWAS tab, diseases are retrieved by keyword search or by traversing a MeSH–based ontology tree; each record displays key metadata (MeSH identifier, PMID, and case/control counts) and an interactive chart that ranks the 10 most strongly associated cellular processes. For the GWAS summary statistics utilized in the DisCP-Atlas, a unique identifier is assigned to each study. In instances where multiple GWAS are employed for the same disease, they are distinctly enumerated using a colon suffix. A results table lists all significant process–trait links, annotated with the leading cell type specificity and top pathway term, and offers direct links to the corresponding single–cell datasets and dedicated process pages for further examination. Each association table includes a “Standardized Cell Type” column generated by Cell Ontology Mapper, which harmonizes heterogeneous cell type labels across datasets and improves cross-study comparability while preserving the original expert annotations.

For single-cell data exploration, users first select a tissue of interest, after which DisCP-Atlas lists all available datasets. Each dataset page displays comprehensive metadata, donor characteristics, sample size, number of detected cellular processes, and data source, together with an interactive UMAP colored by cell type and a pie chart of cell type composition, allowing quick inspection of intratissue heterogeneity. A summary table enumerates all cellular processes defined in the tissue, annotated with their predominant cell types and top enriched pathways. Selecting a process opens a dedicated view that reports its disease associations (with *P*-values and *E*-scores), visualizes activity patterns through UMAP and violin plots, and lists all constituent genes with contribution scores. Functional annotations are summarized in an interactive bar plot of the leading GO, KEGG, and Reactome terms, and full enrichment tables can be downloaded.

At the gene level, users can browse or search the complete gene catalog. Each gene page provides basic identifiers (Ensembl ID, gene type, genomic coordinates, synonyms) and the number of modules in which the gene occurs; it also displays the gene’s significance scores across modules and offers one–click navigation to the relevant process pages. Finally, an intuitive enrichment tool accepts user–defined gene lists, identifies cellular processes significantly over–represented in those genes, and presents the results in an interactive bar plot alongside a downloadable statistics file. DisCP-Atlas currently focuses on gene and cellular-process level, and SNP-to-gene mapping, particularly for noncoding variants, relies on external quantitative trait locus (QTL) and epigenomic resources. Accordingly, we provide links to dbSNP [48], Open Targets [49], and TargetGene [50], so that users can identify candidate target genes and then explore their implicated cellular processes within DisCP-Atlas.

### Case study: identifying schizophrenia–relevant cellular processes

**Figure 3.**
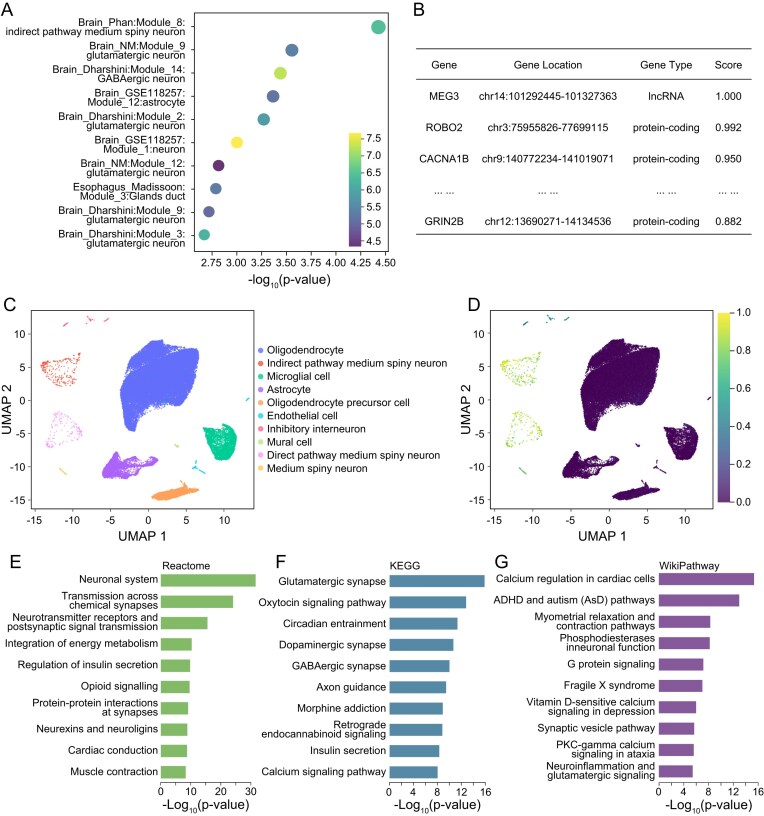
Case study of schizophrenia using DisCP–Atlas. (**A**) Dot plot showing the 10 cellular processes most significantly associated with schizophrenia. (**B**) Table highlighting the key genes within the top schizophrenia–associated cellular process. (**C**) UMAP visualization colored by annotated cell types, illustrating cellular heterogeneity within the single–cell dataset. (**D**) UMAP visualization colored by activity levels of the top schizophrenia–associated cellular process. Bar plots presenting the top enriched Reactome (**E**), KEGG (**F**), and WikiPathways (**G**) terms for the top cellular process.

Schizophrenia is a chronic psychiatric disorder that affects ~1% of the population and, according to large twin–study meta–analyses, has a broad–sense heritability of ~0.8 [51]. To illustrate how DisCP–Atlas pinpoints disease–related biology, we analyzed the schizophrenia GWAS contained in the atlas (33 640 cases, 43 456 controls; GWAS Catalog). From the GWAS tab, users enter “schizophrenia” or navigate the MeSH tree to open the trait page with ID DisCP0636:1, which summarizes study metadata and displays an interactive dot plot of the 10 most strongly associated cellular processes. The top signal (lowest *P*-value) is Module 8 from the Brain_Phan single–cell dataset (Fig. 3A). Selecting this link takes users to the module page, which details the striatal dataset (12 donor samples) and presents an interactive UMAP colored by cell type, a pie chart of cell–type composition, and tables listing enriched pathways, constituent genes with contribution scores, and all significant disease links (Fig. 3C). Together, these views allow rapid inspection of brain–specific cellular programs most relevant to schizophrenia.

UMAP and violin plots indicate that Module_8 exhibits the strongest activity in neuronal populations, especially medium spiny neurons (direct and indirect pathways) and inhibitory interneurons (Fig. 3D). Pathway enrichment analysis further links Module_8 to critical neuronal functions: Neuronal System, Chemical Synaptic Transmission, and Neurotransmitter Receptor Signaling (Reactome; Fig. 3E); Glutamatergic Synapse, Dopaminergic Synapse, and Axon Guidance (KEGG; Fig. 3F); Phosphodiesterase in Neuronal Function, Neuroinflammation, and Glutamatergic Signaling (WikiPathways; Fig. 3G). These associations align closely with accumulating evidence that schizophrenia risk genes are enriched in neuron-specific pathways, particularly those governing synapse formation and plasticity in the mature brain [52, 53]. Single–cell transcriptomic analyses have also demonstrated that schizophrenia–associated risk genes are predominantly expressed in striatal medium spiny projection neurons and cortical excitatory neurons [54]. Mechanistic studies further indicate that these genes disrupt synaptic architecture and plasticity, for instance, overexpression of the schizophrenia–associated gene C4 impairs intracellular trafficking of postsynaptic GluR1–containing AMPA receptors, leading to pathological spine loss and synaptic deficits in cortical neurons [55].

Further support for Module_8’s association with schizophrenia comes from its constituent genes, many of which are implicated in disease pathophysiology (Fig. 3B), including *MEG3* (an long non-coding RNA (lncRNA) upregulated in peripheral blood of female schizophrenia patients) [56], *ROBO2* (a GWAS-identified susceptibility gene) [57], *CACNA1B* (a voltage-gated calcium channel subunit critical for synaptic plasticity) [58], and *GRIN2B* (a risk locus in GWAS/transcriptomic meta-analyses encoding the NR2B subunit of the NMDA receptor) [59]. DisCP–Atlas also provides detailed, gene–level information, accessible via links to dedicated gene pages. This allows exploration of other cellular processes associated with each gene. In addition to the schizophrenia case study, further examples of cellular process–disease associations are provided in Supplementary Figs S2 and S3. These include the relationship between heart failure and cardiomyocytes in the heart, irritable bowel syndrome and colonic epithelial cells, glaucoma and Müller cells in the retina, systemic lupus erythematosus and interferon-related functions, and alcoholic liver damage and cholangiocytes in the liver. A nonexhaustive list of additional cellular process–disease associations is available in Supplementary Table S3. Collectively, these examples demonstrate the broad applicability of DisCP-Atlas across diverse disease categories and tissue types, highlighting the utility of DisCP–Atlas for dissecting complex disease pathogenesis through the systematic integration of genetic and single–cell transcriptomic data.

## Discussion

GWAS have identified numerous risk loci and candidate genes for complex human diseases [1–4, 60]. However, the specific cell types and cellular processes through which these genes exert their effects remain largely unclear [6, 8]. Elucidating these cellular contexts of diseases is critical for translating genetic associations into therapeutics. The development of scRNA-seq has enabled gene expression profiling at cellular resolution, providing a powerful resource for functionally annotating GWAS results. Leveraging the established sc-linker framework [38], DisCP-Atlas systematically identifies cellular processes from single-cell transcriptomic data and integrates them with complex diseases based on their underlying genetic architectures, thus revealing disease-relevant cellular processes.

DisCP-Atlas adopts the sc-linker framework, which has been benchmarked in its original publication and shown to recover gold-standard enrichments. To showcase sc-linker is able to capture gold-standard functions, we performed analysis using well-characterized hematological traits that are not part of our disease catalog. For monocyte count, the top processes localized to classical and nonclassical monocytes and highlighted neutrophil degranulation pathways (Blood_Rabadam, Module_3; *E*-score = 17.20; *P*-value = 7.13 × 10^−7^). For lymphocyte count, leading processes mapped to naive CD4⁺ T cells and B-cell subsets with translation-elongation pathways enriched (Blood_Rabadam, Module_11 and Module_14; *E*-score = 13.25 and 10.33; *P*-value = 3.37 × 10^−7^ and 4.66 × 10^−8^). These results recapitulate established biology and support the validity of our cellular-process enrichment readouts. Detailed statistics are provided in Supplementary Table S5.

DisCP-Atlas offers significant advantages compared with existing disease-cell type databases. Traditional resources commonly rely on discrete clustering and predefined cell type markers, which do not fully capture the continuous and dynamic nature of cellular states [15]. Cells naturally exist along gradients, dynamically responding to environmental signals, developmental stages, and disease processes [61, 62]. A cellular process-centric approach effectively captures this biological complexity, whereas traditional cell type classifications may artificially segregate cells into discrete groups, obscuring important functional variability [40, 63]. Moreover, many current databases utilize enrichment- or overexpression-based methods that fail to consider the polygenic nature of complex diseases. DisCP-Atlas addresses these limitations by systematically integrating single-cell transcriptomic data with GWAS signals using the widely adopted sc-linker framework, thereby accounting for linkage disequilibrium structure and providing more precise and biologically relevant mappings of cellular contexts to disease mechanisms.

Despite its advantages, DisCP-Atlas has several limitations that highlight important directions for future development. First, we did not utilize prior knowledge to restrict analyses to tissues previously associated with each disease. While this strategy helps uncover novel biological connections, it may also introduce confounding associations or results with uncertain biological interpretations. For example, our analysis identified a novel connection between irritable bowel syndrome and brain-specific cellular processes, consistent with emerging evidence supporting the gut–brain axis [64]. Future improvements may incorporate prior biological knowledge to refine disease-specific tissue associations, improving interpretability and accuracy.

In addition, the current version of DisCP-Atlas primarily includes GWAS data derived from European-ancestry populations, limiting its applicability across diverse populations. Incorporating GWAS datasets from non-European populations in future updates will significantly broaden the database’s global utility. Furthermore, although we included a wide range of single-cell RNA-seq datasets, certain human tissues, developmental stages, and disease conditions remain underrepresented. To address this, future updates will integrate additional single-cell datasets covering a greater diversity of biological conditions, as well as potentially extending to spatial transcriptomics, thereby providing a more comprehensive understanding of disease-associated cellular processes.

In summary, DisCP-Atlas systematically integrates single-cell RNA-seq datasets with GWAS summary statistics, establishing a comprehensive resource linking cellular processes to complex diseases. By clarifying the cellular contexts underlying genetic associations, DisCP-Atlas facilitates the biological interpretation of disease mechanisms, promotes mechanistic understanding, and supports the identification of potential therapeutic targets. Future updates and ongoing expansions of this resource will further strengthen its value as an essential research resource for the biomedical research community.

## Supplementary Material

gkaf1129_Supplemental_Files

## Data Availability

All relevant data are available through the DisCP-Atlas website (https://www.discpatlas.net). The UK Biobank data used in this study were obtained under application number 144904. While no new experimental data were generated, extensive analysis was performed on publicly available single-cell and GWAS datasets to construct the DisCP-Atlas resource.
