# Supplementary material for: DisCP-Atlas: a comprehensive resource mapping cellular processes to complex diseases
Source: Nucleic Acids Res. 2025 Nov 3;54(D1):D1376–86. doi: 10.1093/nar/gkaf1129 (PMC12807598; doi:10.1093/nar/gkaf1129)
Supplement: gkaf1129_Supplemental_Files [file gkaf1129_supplemental_files.zip › Supplementary_Figure.pdf]

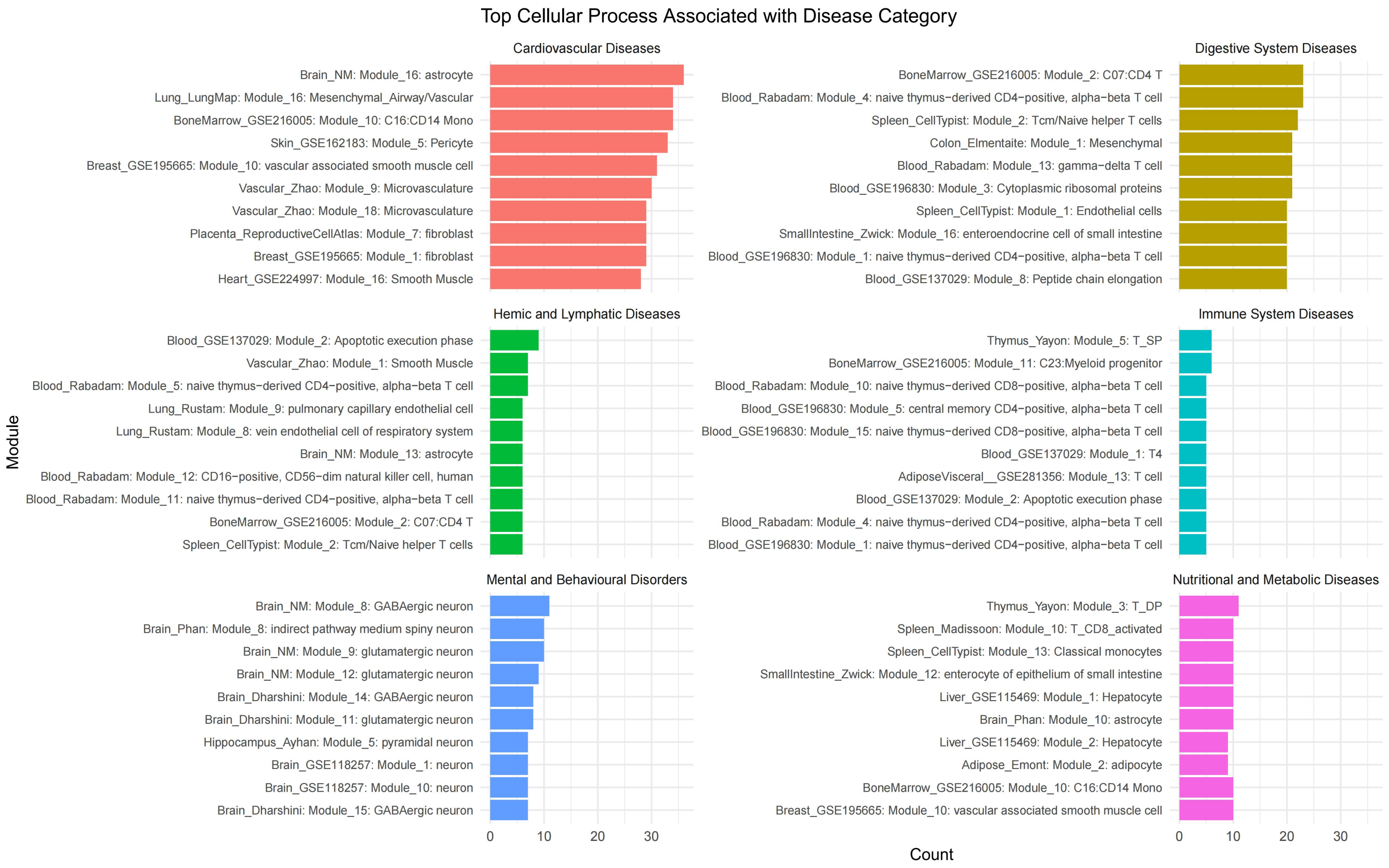

Supplementary Figure 1. Top Cellualr Processes Associated with Different Disease Categories.

Heart Failure

SingleCell	ModuleName	Escore	pValue	Enriched Cell Type	Enriched Pathway
Heart_ GSE224997	Module_18: Cardiomyocyte	11.22	2.87e-02	Cardiomyocyt e	Electron transport chain: OXPHOS system in mitochondria
Heart_Linna	Module_13: pericyte	7.65	4.30e-02	pericyte; smooth muscle cell	cGMP-PKG signaling pathway
Heart_Linna	Module_7: fibroblast	10.99	3.68e-02	fibroblast	Extracellular matrix organization
…	…	…	…	…	…

Heart_GSE224997 Module_18: Cardiomyocyte Heart_Linna Module_13: pericyte

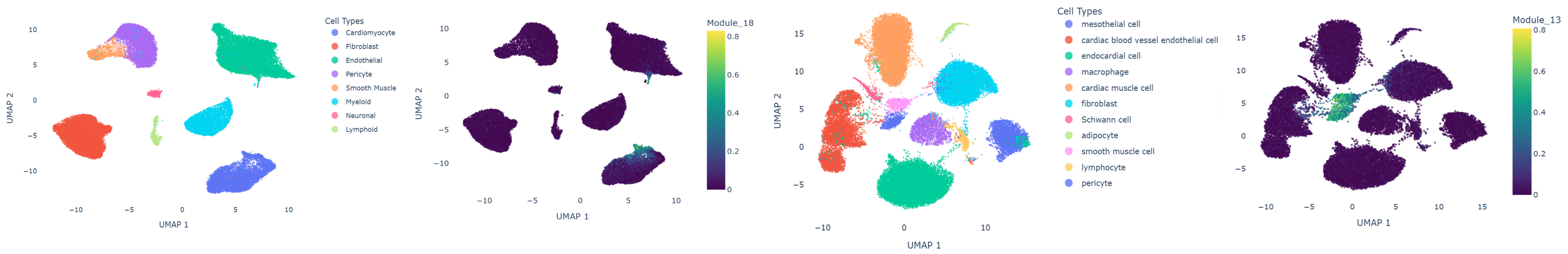

Glaucoma

SingleCell	ModuleName	Escore	pValue	Enriched Cell Type	Enriched Pathway
Retina_E-MTAB-7316	Module_14: Transcriptional regulation by RUNX1	8.34	1.32e-02	Shared Program	Transcriptional regulation by RUNX1
Retina_ GSE268630	Module_17: Muller cell	4.37	1.47e-02	Muller cell	Pleural mesothelioma
Retina_ GSE268630	Module_2: retinal progenitor cell	7.22	1.04e-02	retinal progenitor cell	Collagen formation
…	…	…	…	…	…

Retina_E-MTAB-7316 Module_14: Transcriptional regulation by RUNX1 Retina_GSE268630 Module_2: retinal progenitor cell

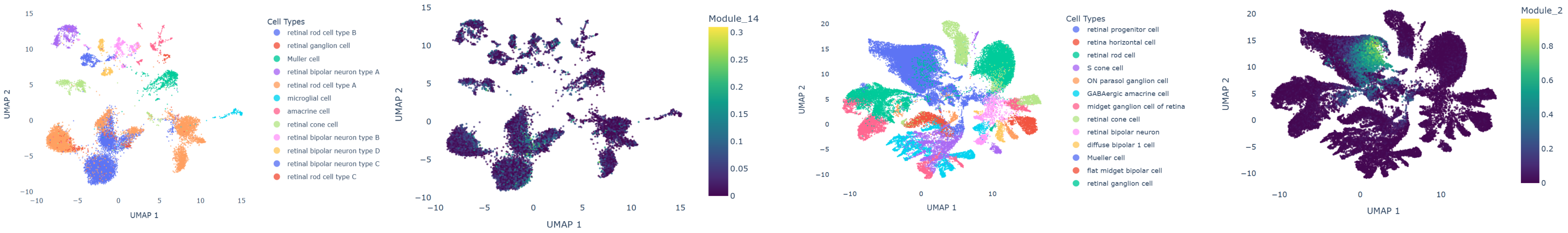

Irritable bowel syndrome

SingleCell	ModuleName	Escore	pValue	Enriched Cell Type	Enriched Pathway
Colon_ Elmentaite	Module_4: Epithelial	4.90	2.60e-02	Epithelial	Peptide chain elongation
Gut_ EGAD000010 10074	Module_15: enterocyte	4.76	2.16e-02	enterocyte	Mineral absorption
Ileum_Martin	Module_5: T cells	11.70	1.52e-02	T cells	Eukaryotic Translation Elongation
…	…	…	…	…	…

Colon_Elmentaite Module_4: Epithelial Gut_EGAD00001010074 Module_15: enterocyte

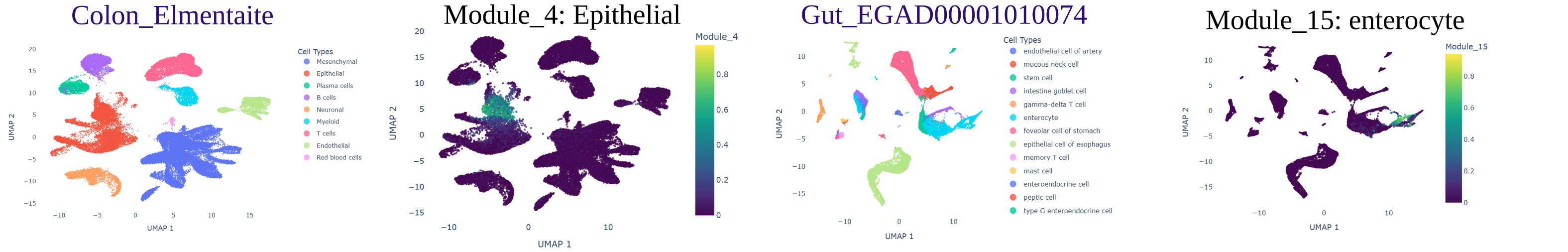

Supplementary Figure 2. Extra cellular process-disease examples in DisCP-Atlas

Systemic lupus erythematosus

SingleCell	ModuleName	Escore	pValue	Enriched Cell Type	Enriched Pathway
Blood_ GSE196830	Module_14: plasmablast	14.03	2.89e-02	plasmablast; plasmacytoid dendritic cell; memory B cell; transitional stage B cell; hematopoietic precursor cell; CD4-positive, alpha-beta T cell	Protein processing in endoplasmic reticulum
Blood_ Rabadam	Module_18: Interferon Signaling	13.59	1.27e-02	Shared Program	Interferon Signaling
Blood_ GSE196830	Module_5: central memory CD4-positive, alpha-beta T cell	10.15	2.88e-02	central memory CD4-positive, alpha-beta T cell; CD4-positive, alpha-beta T cell; effector memory CD4-positive, alpha-beta T cell; platelet	Signaling by Interleukins
...	...	...	...	...	...

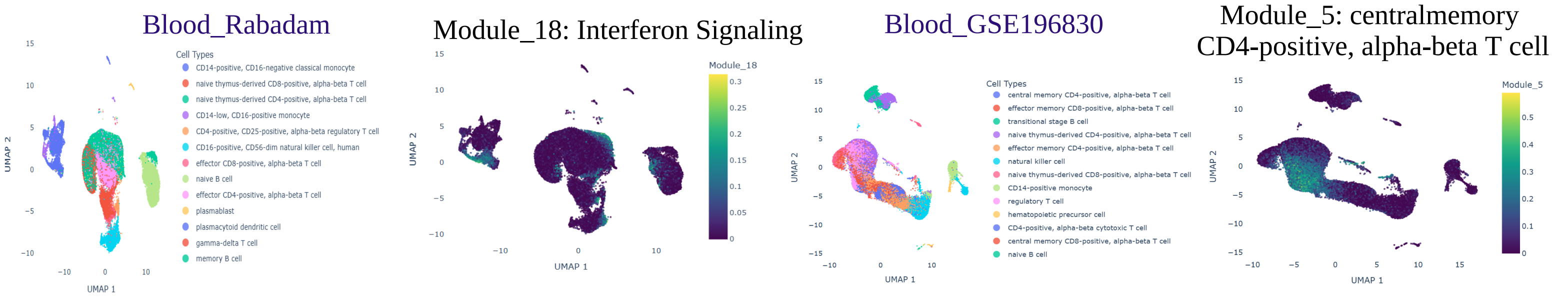

Alcoholic liver damage

SingleCell	ModuleName	Escore	pValue	Enriched Cell Type	Enriched Pathway
Liver_ GSE115469	Module_9: Cholangiocyte	38.07	3.02e-02	Cholangiocyte; Stellate	Diabetic cardiomyopathy
Liver_ GSE180665	Module_9: B cell	28.04	4.46e-02	B cell; hepatocyte; endothelial cell; cholangiocyte	Complement and coagulation cascades
...	...	...	...	...	...

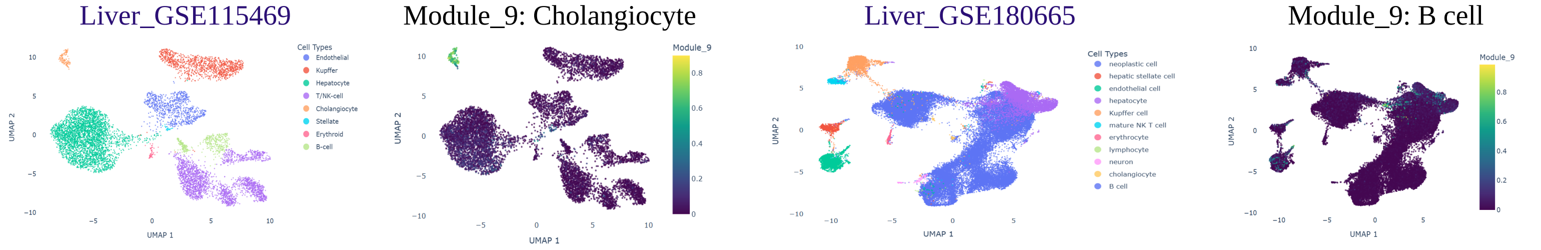

Supplementary Figure 3. Extra cellular process-disease examples in DisCP-Atlas
